# Closed drainage versus non-drainage for single-level lumbar discectomy: *a prospective randomized controlled study*

**DOI:** 10.1186/s12891-020-03504-x

**Published:** 2020-07-22

**Authors:** Hua Guo, Biao Wang, Zhaohua Ji, Xi Gao, Yuting Zhang, Li Yuan, Dingjun Hao

**Affiliations:** 1 Department of Spine Surgery, Xi’an Jiaotong University College of Medicine, Honghui Hospital, Xi’an, Shaanxi China; 2 Department of epidemiology, Shaanxi Provincial Key Laboratory of Free Radical Biology and Medicine, The Ministry of Education Key Laboratory of Hazard Assessment and Control in Special Operational Environment, School of Public Health, Air Force Medical University, Xi’an, Shaanxi China; 3 Department of Intensive Care Unit, Xi’an Jiaotong University College of Medicine, Honghui Hospital, Xi’an, Shaanxi China; 4 Department of Computed Tomography, Xi’an Jiaotong University College of Medicine, Honghui Hospital, Xi’an, Shaanxi China

**Keywords:** Lumbar discectomy, Wound drainage, Randomized controlled study

## Abstract

**Background:**

Postoperative epidural haematoma and wound infection can cause devastating neurological damage in spinal surgery. Closed drainage is a common method to prevent epidural haematoma, infection and related neurological impairment after lumbar decompression; however, it is not clear whether drainage can reduce postoperative complications and improve clinical efficacy. This randomized study aims to explore the role of closed drainage in reducing postoperative complications and improving the clinical efficacy of single-level lumbar discectomy.

**Methods:**

A total of 420 patients with single-level lumbar disc herniation were finally included in this study (169 females and 251 males, age 50.0 ± 6.4 years). A total of 214 patients were randomly assigned to the closed drainage group, and 206 patients were assigned to the non-drainage group. The incidence of postoperative fever, symptomatic epidural haematoma, wound infection and the need for revision surgery were compared between the two groups by the chi-square test or Fisher’s exact test. The visual analogue scale (VAS) and oswestry disability index (ODI) were used to evaluate the improvement of pain relief and the recovery of lumbar function. The VAS and ODI scores were compared between the two groups using t tests.

**Results:**

The complications of the two groups were compared and analysed. There was only a statistically significant difference in the postoperative fever rate (*p* = 0.022), as the non-drainage group had a higher fever rate, but there were no significant differences in the rates of symptomatic epidural haematoma, wound infection or revision operation (*p* > 0.05). After concrete analysis, for the rate of fever less than 38.5 degrees, there was a statistically significant difference (*p* = 0.027), but there was no significant difference when the fever was greater than 38.5 degrees (*p* > 0.05). When comparing the VAS scores of the operation area on the first day after the operation, the pain relief in the closed drainage group was significantly better than that in the non-drainage group, with scores of 5.1 ± 0.8 and 6.0 ± 0.7, respectively (*p* < 0.001). However, there was no significant difference between the two groups in the other VAS scores of operation areas, the VAS scores of the lower extremity, or the ODI scores (*p* > 0.05).

**Conclusions:**

For single-level lumbar discectomy, closed drainage is beneficial for reducing postoperative low-grade fever and relieving pain in the operation area in the very early postoperative stage. However, drainage does not have a significant impact on reducing the incidence of postoperative complications or improving clinical efficacy.

**Trial registration:**

Current Controlled Trials ChiCTR1800016005, May/06/2018, retrospectively registered.

## Background

Surgeons have been using postoperative drainage for generations [1]. The use of drains in medicine has been dated back as far as ancient Egyptians. Hippocrates was the first to describe the use of hollow tubes for the drainage of surgical wounds (~ 460–377 B.C.) [2]. Closed drainage has been commonly used in orthopaedic surgery, particularly in spine surgery, primarily to prevent the formation of hematoma [1, 3–5]; considering that hematoma provides an excellent culture medium for infections, haematoma evacuation is considered advisable to prevent postoperative infections [3, 6].

However, despite the long history of drain usage, the use of drainage after surgery has rarely been supported in the scientific literature. Drains have not been shown to provide benefits with respect to the rates of infection or haematoma in fracture or trauma surgeries [7, 8]. Some retrospective studies have suggested that drains may be unnecessary in spine operations [3]. It is difficult to dispute the seemingly logical concept of wound drainage. The empirical use of drains to avoid postoperative haematoma formation has been called into question [1]. In spine surgery, postoperative epidural haematomas and wound infections can have devastating neurological impairment [9–14]. Mohi et al. [15] and Mirzai et al. [6] reported that epidural haematomas on the first postoperative day after lumbar disc surgery occurred as frequently as in 86 to 89% of patients according to magnetic resonance imaging (MRI) scans. For such a high incidence of haematoma, however, the vast majority of haematomas do not have any effect on patients, and symptomatic epidural haematomas are uncommon after lumbar disc surgery. The incidence of postoperative symptomatic epidural haematomas is only 0.2–2.9% in all spine operations requiring revision surgery [3, 6, 16].

As a result, closed drainage is commonly used for the prevention of postoperative haematoma, infection, and associated neurologic impairment after lumbar decompression, but it remains unclear whether closed drainage reduces postoperative complications and improves clinical outcomes. To answer these questions, this prospective, randomized study was designed to determine the efficacy of prophylactic closed drainage in improving clinical outcomes after single-level lumbar discectomy.

## Methods

Patients with single-level lumbar disc herniation were allocated to either the closed drainage group or the non-drainage group during a 3-year period (between March 2012 and March 2015). The ethics committee of our medical centre approved the study protocol, and patients provided written informed consent before participation. The authors confirm that all ongoing and related trials for this intervention are registered. Since our research centre had not registered before the start of the study, there was a delay in registration.

The diagnosis of lumbar disc herniation was based on preoperative symptoms, clinical examination, and spinal nerve root compression detected by MRI examination [17]. Indications for lumbar discectomy surgery in disc herniation patients were extensive or unbearable pain radiating down to the lower extremity and/or muscle weakness with ineffective conservative treatment and, in the majority of patients, a positive straight leg raising test with a value of < 60 degrees. Patients may also have a loss of the Achilles reflex, cauda equina syndrome and/or regional sensory loss.

The inclusion criteria were as follows: patients with single-level lumbar disc herniation in accordance with surgical indications. The exclusion criteria were as follows: patients with an abnormal activated partial thromboplastin time (APTT), partial thromboplastin time (PTT) or fibrinogen count; the preoperative use of anticoagulant or antiplatelet drugs; any known bleeding disorder (thrombocytopenia, coagulation factor deficiency); a history of diabetes, senile psychosis, cerebral disease with cognitive damage, malignant tumour, metabolic osteopathy, spinal infection, dermatosis, or cerebrospinal fluid leakage due to dural injury during the operation; and patients who underwent previous surgery on the herniated disc segment or those with other spinal disorders.

**Fig. 1:**
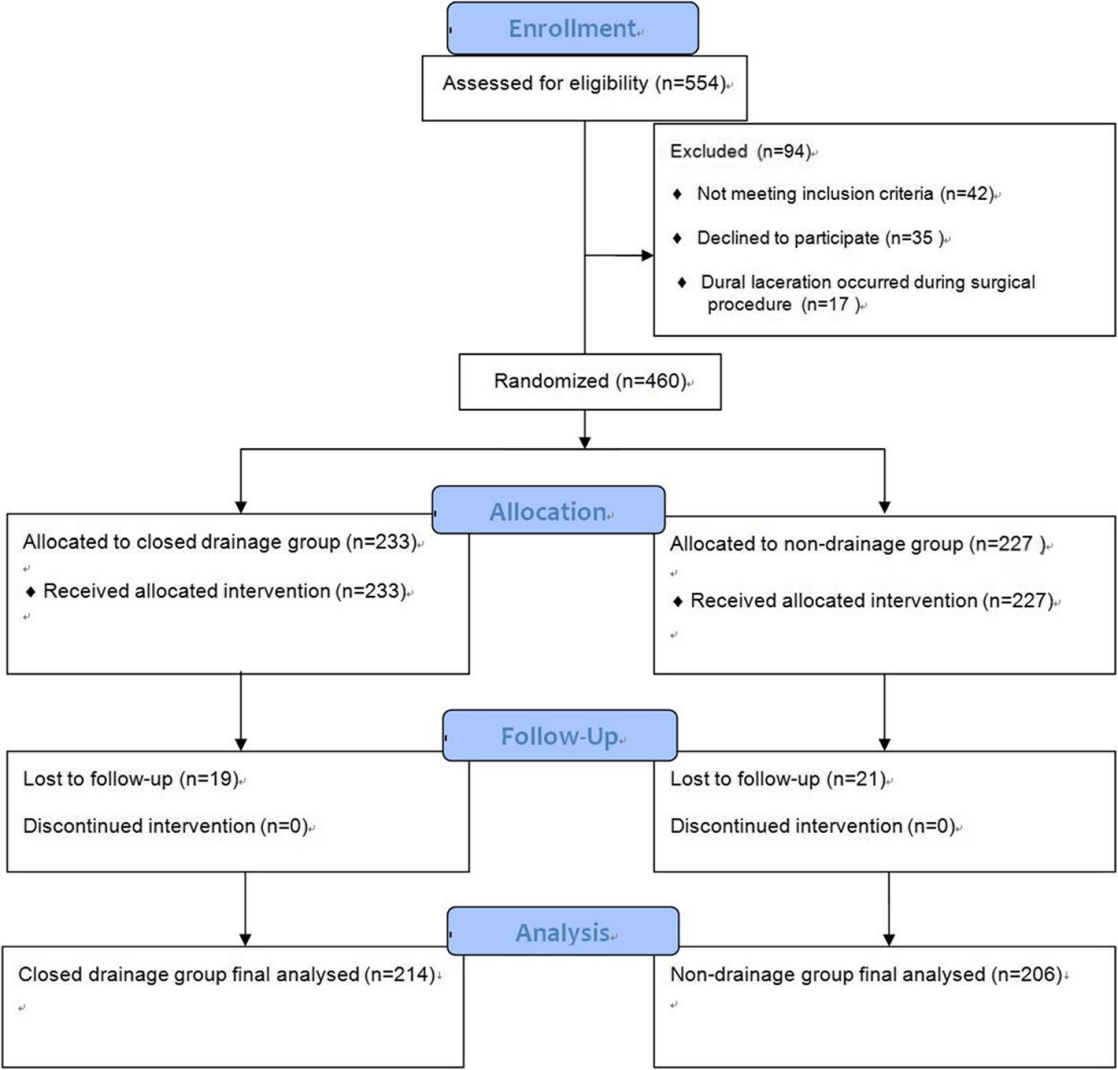
The flowchart. A drawing of the elements of the study design

There were 169 female and 251 male patients included in this study, with a mean age of 50.0 ± 6.4 years (range 27 to 64 years). Preoperative lumbar disc herniation was detected at the L3/L4 level in 39 (9.3%) cases, L4/L5 in 208 (49.5%) cases, and L5/S1 in 173 (41.2%) cases. The patients were randomly assigned to either the closed drainage group (214 patients) or the non-drainage group (206 patients). A schematic diagram of this study design is shown in Fig. 1, with the demographic information about the patients who were present at the final follow-up provided in Table 1.

**Table 1:** Demographic and clinical characteristics of the patients

	Closed drainage group (*n* = 214)	Non-drainage group (*n* = 206)	*p*
Age (yr.)	50.4 ± 6.6	49.6 ± 6.2	0.220
Male to female ratio	122:92	129:77	–
Operation time (min.)	60.6 ± 4.1	60.2 ± 3.9	0.359
Intraoperative blood loss (ml.)	62.7 ± 6.5	62.6 ± 6.2	0.856
Hemorrhagic collection (ml.)	54.2 ± 15.5	–	–

Data are presented as mean ± standard deviation

To estimate the required sample size, we used the epidural haematoma rate according to Kadir Kotil’s study, in which the epidural haematoma rates were 5 and 16.3% in the closed drainage and non-drainage groups, respectively [16]. PASS 11™ software (NCSS, Kaysville, UT) was used to calculate the sample size. The results showed that 179 subjects were needed per group with an α of 0.05 and a power of 90%. In this study, the number of patients in both groups (closed drainage group, 214 patients and non-drainage group, 206 patients) was larger than the required sample size.

Antibiotics were given to all patients prophylactically. Thirty minutes before the incision, systemic prophylactic antibiotic therapy with cefazolin was administered intravenously at a dosage of 2 g every 12 h until 24 h postoperatively. However, clindamycin was used in patients with a positive cephalosporin skin test.

All discectomies were performed by the same surgeon using standard techniques on a virgin (unexplored) single segment. The surgical technique is described as follows. The patient was placed in the prone position after general anaesthesia with endotracheal intubation. Due to the short operation time, no urinary catheterization was performed. A C-arm was used to confirm the position of the herniated disc and make skin markers. A midline skin incision of approximately 3 cm was made according to the surface marker, and then the subcutaneous tissue and the lumbodorsal fascia were cut layer by layer. The paraspinal muscles were then stripped unilaterally in a subperiosteal fashion. After complete exposure, partial hemilaminectomy was performed, and the ligamentum flavum was opened enough to expose the dural sac, the compromised nerve root, and the entire herniated disc. Temporary compression with a gelatine sponge and bipolar electrocoagulation were used for epidural haemostasis when necessary. The disc was then removed with the nerve root carefully retracted medially. The performance of wound closure was the same in both groups except that different drainage methods were used. A gravity drainage device was used in the drainage group.

Two sealed envelopes with one card each were prepared by the research assistant before the operation, and the assistant did not participate in the surgical treatment process. One card read “drainage”, and the other read “no drainage”, which determined whether patients were randomly divided into the “closed drainage group” or the “non-drainage group”. When the discectomy was over, before closing the wound, a nurse randomly selected an envelope to open and told the surgeon whether the card in the envelope read drainage or non-drainage. The random result determined whether the surgeon placed a drainage tube in the patient. If dural tears occurred during surgery, patients were excluded from the study. Although these patients were initially included to participate in the study, they received drains that were excluded from the randomization process and were not included in the final study results. Another scientific assistant completed the collection of clinical data in the two groups. Therefore, the participants, care providers, and assessors were all blinded before grouping. However, it was obvious whether a drainage tube was placed in postoperative patients; at this time the above-mentioned persons could not be blinded.

If the drainage volume was less than 50 ml in the previous 24 h, the drain was removed. All drains were withdrawn 24 or 48 h postoperatively. Before the removal of the drainage tube, all the patients in the drainage group received a few drainage fluid collections for bacterial culture and drug sensitivity tests to detect and treat infection risk factors as early as possible. At the time of drain withdrawal, the amount of haemorrhagic collection was between 28 and 150 ml, with an average of 54.2 ± 15.5 ml.

The postoperative clinical follow-up period was 2 years. The postoperative complications of the two groups (fever, symptomatic epidural haematoma, wound infections, and requiring revision surgery) were recorded. Pain intensity was evaluated by the visual analogue scale (VAS). Functional ability was measured for all of the patients using the oswestry disability index (ODI). The postoperative results of pain and functional ability as measured by the VAS and ODI were obtained at the time of discharge. The lower extremity VAS score and ODI score were evaluated preoperatively, postoperatively, and at the last follow-up. The VAS score of the operation area was evaluated preoperatively; on day 1, week 1, week 2, and month 1 postoperatively; and at the last follow-up.

The rates of postoperative complications were compared between the two groups using the chi-square test or Fisher’s exact test. The VAS and ODI scores were compared between the two groups using t tests. Data were analysed using SPSS version 19.0 statistical software (SPSS, Chicago, IL, USA). Quantitative results are presented as the mean ± standard deviation. Bilateral *p* < 0.05 was considered statistically significant.

## Results

### Demographic characteristics of the patients

With respect to the mean age, operation time, and intraoperative blood loss, the differences between patients in the closed drainage group and the non-drainage group were not statistically significant (*p* > 0.05) (Table 1). The patients in the two groups were homogenous and comparable.

### Primary results

In both groups, a total of 98 patients (23.3%) developed fever postoperatively. The difference in postoperative fever between the closed drainage group (18.7%) and the non-drainage group (28.2%) was statistically significant (*p* < 0.05). After concrete analysis, in the closed drainage group, 38 (95.0%) of the 40 febrile patients had a fever less than 38.5 degrees, and the remaining 2 (5.0%) patients had a fever greater than 38.5 degrees. In the non-drainage group, 55 (94.8%) of the 58 febrile patients had a fever less than 38.5 degrees, and the remaining 3 (5.2%) patients had a fever greater than 38.5 degrees. For the rate of fever less than 38.5 degrees, there was a statistically significant difference between the two groups (*p* < 0.05). However, there was no significant difference between the two groups in terms of the rate of fever greater than 38.5 degrees (*p* > 0.05).

**Fig. 2:**
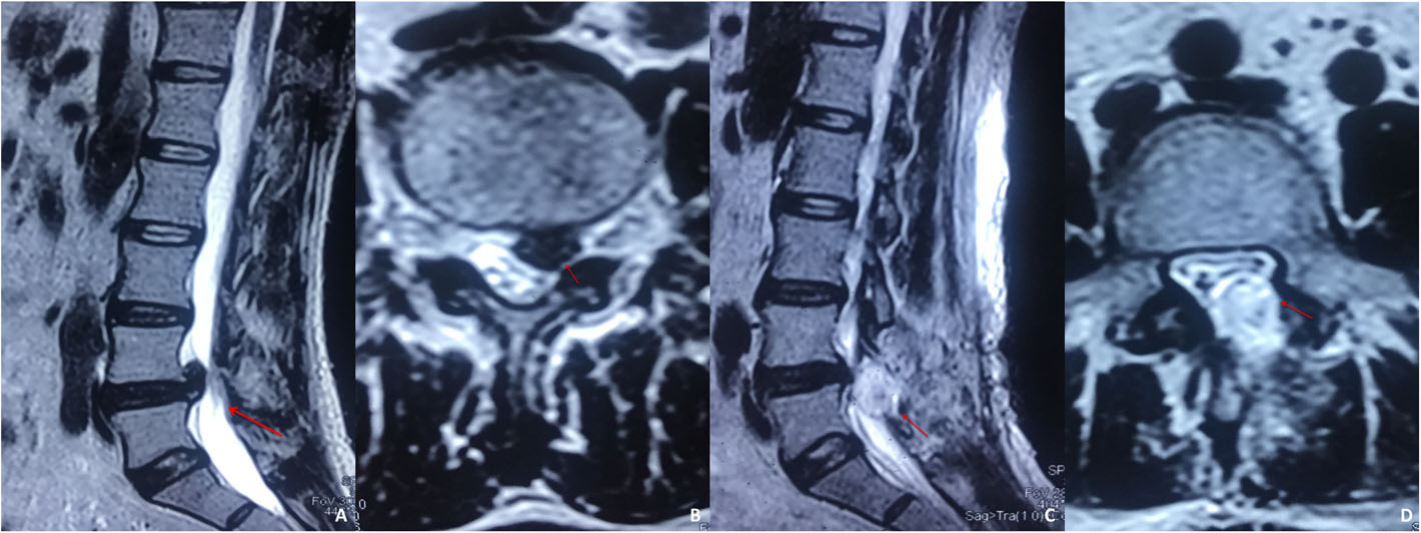
Only one patient had symptomatic epidural haematoma. A 37-year-old male with a herniated disc at the L4/5 segment required discectomy treatment. (**a**) Sagittal MRI showed the L4/5 segment with a substantially herniated disc. (**b**) Transverse MRI showed herniated nucleus pulposus tissue, resulting in compression of the left nerve root. (**c d**) The symptoms of left lower limb pain recurred after discectomy. The sagittal and transverse MRI sections showed haematoma formation, and the dural sac and left nerve root were obviously compressed

There was no significant difference in symptomatic epidural haematoma when the two groups were compared. Only one patient with L4/5 disc herniation in the non-drainage group had recurrence of lower extremity symptoms on the second day after surgery (Fig. 2). The MRI examination revealed the formation of epidural haematoma, resulting in nerve compression and recurrence of lower extremity symptoms. The patient’s symptoms were improved by removing the epidural haematoma through revision surgery. There was no difference in the risk of wound infection, as none of the 420 patients had wound infections.

In the closed drainage group, stump rupture was found in one patient after removal of the drainage tube. Then, revision surgery was performed to remove the stump of the drain left inside the patient. Consequently, a total of two patients underwent revision surgery, one in each group. The difference in the revision operation rate between patients in the closed drainage group (0.5%) and the non-drainage group (0.5%) was not statistically significant (*p* > 0.05). The primary results are listed in Table 2.

**Table 2:** Summary of the primary clinical results

	Closed drainage group	Non-drainage group	*p*
Fever	40	58	0.022^a^
< 38.5 °C	38	55	0.027^a^
≥ 38.5 °C	2	3	0.308^b^
SEH	0	1	0.491^b^
Infection	0	0	–
Revision surgery	1	1	0.501^b^

*Abbreviation*: *SEH* symptomatic epidural hematomas

^a^chi square test

^b^Fisher exact test

### Follow-up results

The follow-up results are presented in Tables 3, 4 and 5. All patients underwent a two-year follow-up period. Preoperatively, postoperatively, and at the last follow-up, there were no significant differences in the lower extremity VAS scores or the ODI scores when the two groups were compared (*p* > 0.05). Similarly, preoperatively, on week 1, week 2, and month 1 postoperatively, and the last follow-up, there was also no significant difference in the operation area VAS scores when the two groups were compared (*p* > 0.05). Only when the day 1 postoperative operation area VAS scores were compared was the closed drainage group better than the non-drainage group, with a significant difference (*p* < 0.05).

**Table 3:** Summary of the operation area VAS follow-up results

	Closed drainage group	Non-drainage group	*p*
Preoperative	2.2 ± 0.8	2.0 ± 0.8	0.143
Day 1	5.1 ± 0.8	6.0 ± 0.7	0.000
Week 1	3.9 ± 0.8	4.0 ± 0.8	0.105
Week 2	2.9 ± 0.9	3.1 ± 0.7	0.127
Month 1	1.3 ± 0.6	1.4 ± 0.7	0.139
Last follow-up	0.8 ± 0.6	0.9 ± 0.6	0.215

Data are presented as mean ± standard deviation

*Abbreviation*: *VAS* visual analog scale

**Table 4:** Summary of the lower extremity VAS follow-up results

	Closed drainage group	Non-drainage group	*p*
Preoperative	7.6 ± 0.8	7.7 ± 0.7	0.179
Postoperative	2.7 ± 0.7	2.8 ± 0.7	0.134
Last follow-up	2.0 ± 0.7	2.1 ± 0.6	0.128

Data are presented as mean ± standard deviation

*Abbreviation*: *VAS* visual analog scale

**Table 5:** Summary of the Oswestry Disability Index follow-up results

	Closed drainage group	Non-drainage group	*p*
Preoperative	59.9 ± 4.1	59.8 ± 4.2	0.736
Postoperative	32.4 ± 4.0	32.8 ± 3.9	0.348
Last follow-up	22.5 ± 3.1	22.8 ± 2.7	0.298

Data are presented as mean ± standard deviation

## Discussion

Closed drainage has long been used following extensive spinal surgery [12–14]. In contrast, the use of drains after single-level disc surgery remains controversial [3, 18]. A prospective, randomized clinical study of 50 single-level lumbar disc surgeries reported by Mirzai et al. [6] indicated that inserting a drain decreased the incidence of haematoma detected by MRI on the first postoperative day, as 89% of patients without a drain had haematomas, and only 36% of patients with a drain had haematomas. This means that the rate of absence of haematoma increased from 11 to 64% after using a drain. In our study, due to the limitations of the study conditions, we did not perform postoperative MRI examinations to compare the incidence of haematoma in both groups. We studied the occurrence of postoperative fever and symptomatic haematoma in the patients. The fever rate in the drainage group (18.7%) was lower than that in the non-drainage group (28.2%). After statistical analysis, we found that the difference in the fever rate was mainly due to the difference between the two groups of patients with low fever. It is well known that low fever is often caused by the absorption of haematoma after surgery. Therefore, the low fever rate may be because the incidence of postoperative haematoma in the drainage group was lower than that in the non-drainage group, and there was less absorption of the haematoma. Symptomatic haematoma occurred in only 1 patient in the two groups, and there was no statistically significant difference in the incidence of symptomatic haematoma between the two groups. Therefore, we believe that drainage may be beneficial in preventing the formation of haematoma after single-level lumbar discectomy, but we do not think there is any significance for reducing the incidence of symptomatic haematoma.

With the progression of medical technology and the increase in aseptic concepts, the infection rate of spine surgery has become increasingly lower [19]. Dimick et al [20] calculated that 5036 patients undergoing spinal surgery would need to be enrolled to show a reduction in the infection rate from 2 to 1%. For single-level lumbar discectomy, a less traumatic surgery, the infection rate is even lower. In 1996, Payne et al [2] randomized 200 single-level lumbar laminectomy patients into two groups based on the presence or absence of a drain and found that 2 of 103 patients (1.9%) with a drain and 1 of 97 patients (1.0%) without a drain had the complication of wound infection. In 2010, Kanayama et al [3] retrospectively reviewed 560 patients who underwent single-level lumbar decompression or discectomy; 298 patients received drains, and the remaining 262 patients did not. As a result, the infection rate was 0, and none of the 560 patients had wound infections. Both authors concluded that closed drainage provided no benefit with respect to rates of infection. Similar results were achieved in our study, with no infection in either of the two groups. Although there was a difference in the postoperative fever rate between the two groups during the first week after the operation, fevers were generally non-infectious fevers, regardless of whether they were low- or high-grade fevers. This general rule was confirmed to a certain extent by 420 patients without infection.

One patient underwent revision surgery in each of the two groups, and there was no significant difference in the reoperation rate. It can be seen that drainage can reduce postoperative fever, and there is no other reduction in postoperative complications in single-level lumbar decompression patients.

At the two-year follow-up, only when the day 1 postoperative operation area VAS scores were compared were the closed drainage group scores of 5.1 ± 0.8 better than the non-drainage group scores of 6.0 ± 0.7, with a significant difference (*p* < 0.05). The other evaluations of the operation area VAS scores, lower extremity VAS scores, and ODI scores were not significantly different between the two groups. This shows that the insertion of a drainage tube eliminates the bleeding and exudate of the intraoperative area and has the effect of relieving pain in the early postoperative period (postoperative day 1), but long-term observation is of no significance for patients’ pain relief and functional recovery.

Although drainage has the advantages of reducing postoperative haematoma formation, reducing postoperative fever, and relieving postoperative pain in the early postoperative period, there are also some disadvantages. It is recognized that drains may provide a conduit for the entry of bacteria. The literature contains conflicting reports on the use of drains [21, 22]. Although there were no infected patients in the drainage group in our study, some drainage systems have been associated with high infection rates [1]. A meta-analysis suggested that drains may do more harm than good and that their only proven benefit is a reduced need for the dressing changes [23]. Furthermore, under the current status of promoting day surgery, patients with drains may not be able to be discharged the day after surgery. This is not conducive to improving medical efficiency.

This study had several limitations. First, we narrowed our study group to single-level lumbar discectomy patients. Because of the lower incidence of spinal epidural haematoma with this procedure compared to other multilevel lumbar procedures, our findings should be applied only to single-level lumbar discectomy and should not be extended to multisegmental disease. Second, due to the limitations of the study conditions, we did not perform postoperative MRI examination to compare the incidence of haematoma in both groups. We speculate that the rate of haematoma formation in the drainage group was lower after drainage in patients with a lower fever rate. Third, some scholars believe that elevated temperature during the first 60 postoperative hours may be associated with pulmonary dysfunction [24]. Although the two groups used the same anesthesia and the operation time was almost the same, there was still the possibility of type I error. Fourth, while the follow-up period of 2 years in the present study was comparable to that used in many literature studies, a longer follow-up regarding epidural fibrosis might enable us to have a better understanding of the long-term effect of using a drain. Furthermore, although this study is by far the largest known randomized controlled study in this area, the number of patients in both groups was small, which may limit the generalizability of the results.

## Conclusions

With this two-year follow-up, prospective, randomized study, we believe that closed drainage can be beneficial to reduce the postoperative fever rate and alleviate operation area pain in the very early postoperative stage. However, closed drainage has no effect on reducing the postoperative occurrence of symptomatic epidural haematoma, wound infection, or the need for revision surgery and has no effect on improving clinical efficacy for single-level lumbar discectomy.

## Data Availability

The datasets used and analysed during the current study are available from the corresponding author on reasonable request.

## Abbreviations

VAS: Visual analog scale
ODI: Oswestry Disability Index
L: Lumbar
MRI: Magnetic Resonance Imaging
